# Surface Fitting for Quasi Scattered Data from Coordinate Measuring Systems

**DOI:** 10.3390/s18010214

**Published:** 2018-01-13

**Authors:** Qing Mao, Shugui Liu, Sen Wang, Xinhui Ma

**Affiliations:** 1 State Key Laboratory of Precision Measuring Technology and Instruments, Tianjin University, Tianjin 300072, China; maoqing.love@163.com (Q.M.); wang.sen.happy@163.com (S.W.); 2 School of Engineering and Computer Science, University of Hull, Hull HU6 7RX, UK; xinhui.ma@hull.ac.uk

**Keywords:** scattered data, NURBS fitting, resampling, iterative projection optimization, hierarchical fitting

## Abstract

Non-uniform rational B-spline (NURBS) surface fitting from data points is wildly used in the fields of computer aided design (CAD), medical imaging, cultural relic representation and object-shape detection. Usually, the measured data acquired from coordinate measuring systems is neither gridded nor completely scattered. The distribution of this kind of data is scattered in physical space, but the data points are stored in a way consistent with the order of measurement, so it is named quasi scattered data in this paper. Therefore they can be organized into rows easily but the number of points in each row is random. In order to overcome the difficulty of surface fitting from this kind of data, a new method based on resampling is proposed. It consists of three major steps: (1) NURBS curve fitting for each row, (2) resampling on the fitted curve and (3) surface fitting from the resampled data. Iterative projection optimization scheme is applied in the first and third step to yield advisable parameterization and reduce the time cost of projection. A resampling approach based on parameters, local peaks and contour curvature is proposed to overcome the problems of nodes redundancy and high time consumption in the fitting of this kind of scattered data. Numerical experiments are conducted with both simulation and practical data, and the results show that the proposed method is fast, effective and robust. What’s more, by analyzing the fitting results acquired form data with different degrees of scatterness it can be demonstrated that the error introduced by resampling is negligible and therefore it is feasible.

## 1. Introduction

**Figure 1:**
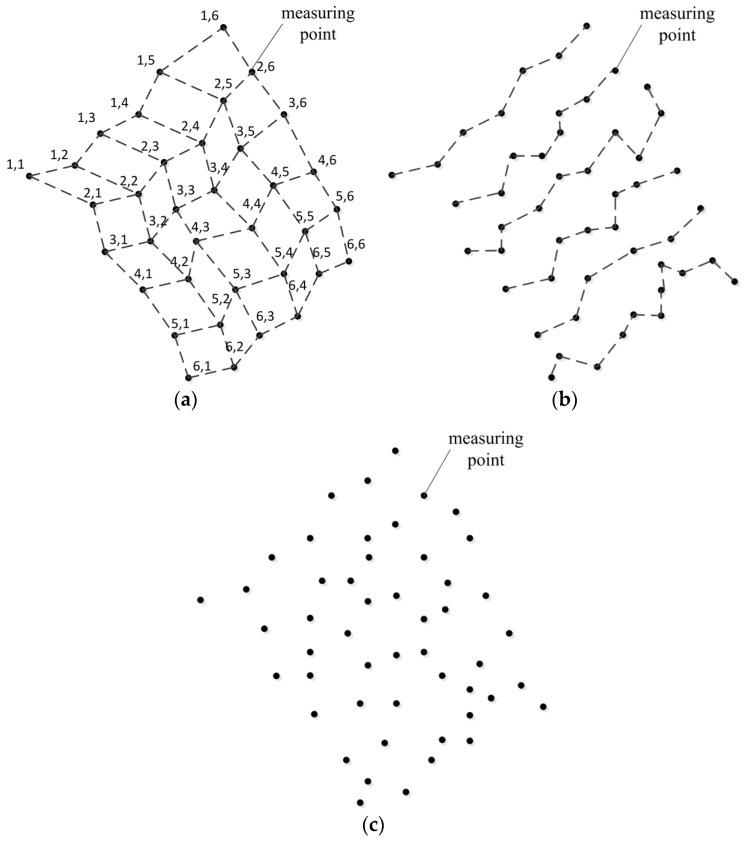
Three different kinds of distribution of data points: (**a**) grid data; (**b**) quasi-scattered data; (**c**) completely scattered data (quasi-scattered data is stored according to the order of measurement which is the main difference from completely scattered data).

The problem of surface fitting appears repetitively in computer aided design, cultural relic representation, reverse engineering, object shape detection and many other fields during the last 20 years or so. Its essence is to build a mathematical model that approximates the object as accurately as possible from measured information. At present, 3D coordinate measuring systems are the main sources of the measured data in these fields [1,2], so finish surface fitting from data acquired from 3D coordinate measuring systems is significant. For now, among all these measuring systems, 3D coordinate measuring machines (CMMs) are representative and mature products which have gained widespread acceptance for their advantages of high accuracy [3]. Besides traditional CMMs, portable 3D vision coordinate measurement machines (PCMMs) such as laser or white light scanners [4,5] and portable light pen 3D vision coordinate measuring systems [6,7], were developed in recent years in order to meet the needs of large-scale on the spot engineering metrology. Generally, the distribution of the measured data acquired from traditional CMMs and the most recently developed PCMMs are neither gridded nor completely scattered [8]. Typically, the data points are scanning points successively sampled from iso-parametric or section curves on a surface, which makes them easily organized into rows [1,2]. In order to describe it clearly, we name this kind of data quasi-scattered data to distinguish it from grid data and completely scattered data in [8]. Figure 1 is a simple sketch and it shows the difference between these three kinds of data. Grid data can easily be organized into rows and columns, and the number of points in each row and column is the same. Quasi-scattered data is scattered in space, but the points are stored according to the order of measurement, so they can easily be organized into rows, but the number of points in each row is different. Completely scattered data has no specific storage order and the points are randomly distributed. In this paper, we only assume that the initial input information is a (possibly massive) set of quasi-scattered 3D points, a typical output obtained from most of the above devices. 

According to the final representation of the fitted surface, the existing related researches can be divided into three categories: fitting methods based on polygonal mesh model [9,10,11,12,13]; fitting methods based on constructive solid geometry (CSG) model [14]; fitting methods based on free-form parametric surface models [15,16,17,18,19,20,21,22]. Non-uniform rational B-spline (NURBS) surface, inheriting all the advantages of B-spline surface, is the most common free-form parametric surface. Most importantly, with the advantages of flexibility and versatility, it provides a good choice for a wide variety of compact and smooth shapes [23,24,25,26,27], so NURBS surface is adopted in this paper.

The problem of NURBS surface fitting from 3D points has been analyzed from several points of view, and for grid data, there are many efficient methods, such as the works related in [19,21,24,25,28,29]. But for scattered data (both quasi-scattered data and completely scattered data), the fitting problem becomes much more difficult because of the limitation that the control points in NURBS surface modeling technique should be organized as a regular grid structure [30]. In [18], a method based on a hierarchical fitting idea is proposed, however, the merged knot vector may make the method face the problem of node redundancy, especially when the number of data points is large and the point distributions of different rows vary a lot. In [28,31] scattered data points are projected to base surfaces to do parameterization according to parameters of the projected points. This kind of methods are useful only when data points can be projected in an unambiguous way [23]. In [30] patch partitioning and polyhedral approximation are adopted to get an organized quadrilateral mesh used as the input of NURBS fitting, however the pre-processing is time consuming and the continuity between different quadrilateral patches is hard to guarantee. In [24] a method combining a hybrid optimization algorithm and iterative scheme (HOAAI) is proposed, which converges rapidly in grid data surface fitting with high accuracy, and when used for scattered data fitting, pre-processing work also need to be done for its strong constraints on the parametrization approach. In order to solve the problem of surface fitting from scattered 3D data, a simulated annealing algorithm [32] and some evolutionary intelligence techniques [23,33,34] were introduced to enhance the robustness. However, these methods are hard to handle. When the measured object is complex or the data size is large, it is not easy to ensure practicability. Under these circumstances, these methods may become low-efficiency because the introduced artificial intelligence techniques are somewhat time-consuming. In [35,36], feature sensitive parameterization is conducted for point-clouds organized in the form of triangulated meshes. They use constant knot vector and allocate more parameter space to area of interest, which results in more control points in the corresponding area. In [35], a kind of area preserving parameterization, stretch minimizing parameterization, is conducted on the image manifold of the original surface. However, the computation of image manifold is not easy and stretch minimizing parameterization requires solving a large non-linear optimization problem, which needs expensive computation. In [36], a hybrid method that combines harmonic mapping and elastic spring is adopted to generate initial parameterization, followed by an adaptive re-parameterization procedure based on relaxation field to refine fitting result. Compared with [35], it requires less computational cost, but the result is not as good geometrically.

In theory, the abovementioned methods provided for completely scattered data fitting are also an available choice for our problem, with no regard to their defects as stated above. If the quasi-scattered data is treated as completely scattered to finish the fitting problem, existing valuable information of measurement order is ignored and extra work must be done for the input data arranging [24,37,38] or initial parameterization [24,28,32,33,34]. For quasi-scattered data which can be easily triangulated, the methods described in [35,36] are applicable. However, the parameterization for quasi-scattered data doesn’t have to be so complicated, because they have already been organized into rows, which is a more efficient structure to manipulate. For now, there is no efficient and general method that can be applied to finish the problem of NURBS surface fitting from quasi-scattered data which appears frequently in reverse engineering applications (see, e.g., [1,2,18]). In this paper, a new method based on resampling, hierarchical fitting and iterative projection is put forward to reconstruct a general NURBS surface from quasi-scattered 3D data. As is known, point projection and data parameterization are two essential problems when iterative projection idea is used for surface fitting. In the provided method, the projection result provides optimized result for subsequent parameterization, and in return the search space of point projection is pointed out accurately by previous parameterization result of the latest iteration. By this way, these two problems are merged together. The main contribution of this paper is that resampling is introduced in surface fitting of quasi-scattered data and a new resampling approach, which makes use of parameterization information of curve fitting and changes of curvature, is proposed. The whole method provided for quasi-scattered data fitting has the advantages of a simple principle, fast computational speed, wide application, and therefore has great utilization value. This paper is structured as follows: the basic definitions, principles and related pervious work for NURBS fitting are introduced in Section 2. Detailed descriptions of the proposed method are provided in Section 3, including the process of the iterative projection optimization scheme, and the implementation of resampling. Section 4 conducts a series of numerical experiments. Finally, discussions and conclusions are provided in Section 5. 

## 2. Pervious Work 

### 2.1. NURBS Curve and Surface

Suppose $U=\{u_{0},u_{1},\ldots ,u_{t-1},u_{t}\}$ is a non-decreasing sequence of real numbers, which is called knot vector and the real numbers $u_{i}$ are its knots. Then the B-spline basis function $N_{i,p}(u)$ of *p* degree (or equivalently, order *p* + 1) can be defined by the recurrence relations, as follows [27,39,40]:

(1) $$N_{i,0}\left(u\right)=\{\begin{array}{cc} 1, & if\;u_{i}\leq u<u_{i+1}\\ 0, & otherwise \end{array}$$

and for 0≤i<r with p≥1:

(2) $$N_{i,p}\left(u\right)=\frac{u-u_{i}}{u_{i+p}-u_{i}}N_{i,p-1}\left(u\right)+\frac{u_{i+p+1}-u}{u_{i+p+1}-u_{i+1}}N_{i+1,p-1}\left(u\right)$$

Here the interval $\left[u_{i},u_{i+p}\right)$ is the support domain of $N_{i,p}(u)$, because the value of $N_{i,p}(u)$ is always zero outside $\left[u_{i},u_{i+p}\right)$. In this paper, NURBS curves and surfaces are defined on the this kind of knot vectors, in which knots distribute non-uniformly and the end knots appear the same times as the order of the B-spline.

Give a knot vector U and a chain of 3D control points $\left\{P_{i},i=0,1,\ldots ,n\right\}$, a NURBS curve with *p* degree is defined as follows [25]:

(3) $$C\left(u\right)=\frac{\sum_{i=0}^{n}N_{i,p}\left(u\right)w_{i}P_{i}}{\sum_{i=0}^{n}N_{i,p}\left(u\right)w_{i}},\;0\leq u\leq 1$$

where $w_{i}$ is the corresponding weight of $P_{i}$, and n+1 is the number of control points. Similarly, give two vectors U, V and a grid of 3D control points $\left\{P_{i,j},i=0,1,\ldots ,n;j=0,1,\ldots ,m\right\}$ which forms a bidirectional net, a NURBS surface with degree (p,q) can be described by:

(4) $$S\left(u,v\right)=\frac{\sum_{i=0}^{n}\sum_{j=0}^{m}N_{i,p}\left(u\right)N_{j,q}\left(v\right)w_{i,j}P_{i,j}}{\sum_{i=0}^{n}\sum_{j=0}^{m}N_{i,p}\left(u\right)N_{j,q}\left(v\right)w_{i,j}},\;0\leq u,v\leq 1$$

### 2.2. Basis of NURBS Curve and Surface Fitting

When the curve degree and the number of control points are already given, the basic process of NURBS curve fitting can be described summarily as follows: (1) parameterize the data points, which means to associate each point with a corresponding parameter; (2) determine the knot vector based on the result of parameterization; (3) calculate the control points based on least squares fitting. The number of control points is ascertained according to a recursive scheme based on the feedback of fitting result which means initializing the number of control points with an empirical value first and subsequently increasing or decreasing it by comparing the fitting precision with given requirement recursively. The curve degree is generally selected according to practical situation. For most applications, a cubic NURBS curve is accurate enough to express the shape of an object.

1. Parameterization of data points. As reported in [22,28,40], the primarily methods to parameterize organized data points are uniform parameterization, which is not recommended when data is unevenly spaced, centripetal parameterization, which is superior when the curvature of the measured object varies intensively, and chord length parameterization, which is most widely used and adopted in the initial part of the proposed method. Taking NURBS curve fitting from data points $Q=\{Q_{i},i=0,1,\ldots ,r\}$ as example, the parameterization result with chord length method $\bar{U}=\{{\bar{u}}_{i},i=0,1,\ldots ,r\}$ is described as follows:
  (5) $$\{\begin{array}{c} {\bar{u}}_{0}=0\\ \begin{array}{cc} {\bar{u}}_{i}={\bar{u}}_{i-1}+\frac{\left|Q_{i}-Q_{i-1}\right|}{\sum_{j=1}^{j=r}\left|Q_{j}-Q_{j-1}\right|}, & i=1,2,\ldots ,r-1 \end{array}\\ {\bar{u}}_{r}=1 \end{array}$$
2. Determination of knot vector. After the parameterization of data points, knot vector $U=\{u_{i},i=0,1,\ldots n+p+1\}$ is initialized based on the parameterization result $\left\{{\bar{u}}_{i},i=0,1,\ldots ,r\right\}$, as follows:
  (6) $$u_{i}=\{\begin{array}{cc} \;0, & i=0,\ldots ,p\\ \left(jt-\left\lfloor jt\right\rfloor \right){\bar{u}}_{\left\lfloor jt\right\rfloor -1}+\left(1-jt+\left\lfloor jt\right\rfloor \right){\bar{u}}_{\left\lfloor jt\right\rfloor }, & i=p+j\;\mathrm{and}\;j=1,\ldots ,n-p\\ \;1, & i=n+1,\ldots ,n+p+1 \end{array}$$
  where n is the number of control points, t is equivalent to (r+1)/(n−p+1) and ⌊jt⌋ is the maximum positive integer that is less than or equal to jt. In this way, at least one parameter ${\bar{u}}_{i}$ is guaranteed in every knot interval, which assures related matrix is well-conditioned.
3. Calculation of control points. When the weights $w_{i}$ are already given, least square curve fitting error can be formulized as
  (7) $$E_{ls}=\sum_{k=0}^{r}{\left|Q_{k}-C\left({\bar{u}}_{k}\right)\right|}^{2}=\sum_{k=0}^{r}{\left(Q_{k}-\sum_{i=0}^{n}R_{i,p}\left({\bar{u}}_{k}\right)P_{i}\right)}^{2}$$

Parameterization result $\left\{{\bar{u}}_{i},i=0,1,\ldots ,r\right\}$ and knot vector $\left\{u_{i},i=0,1,\ldots ,n+p+1\right\}$ are obtained with the method above, $R_{i,p}({\bar{u}}_{k})$ is equal to $N_{i,p}({\bar{u}}_{k})w_{i}/\sum_{i=0}^{n}N_{i,p}({\bar{u}}_{k})w_{i}$ and control points $\left\{P_{i},i=0,1,\ldots ,n\right\}$ are the unknowns. Rewrite Equation (7) as the following matrix form:

(8) $$E_{ls}=\left(P^{T}N^{T}-F^{T}\right)\left(NP-F\right)$$

Here P is the matrix form of control points $\left\{P_{i},i=0,1,\ldots ,n\right\}$, F is the vectorization result of the data points $\left\{Q_{k},k=0,1,\ldots ,r\right\}$, and N is the coefficient matrix composed of basic functions:

$$P=\left[\begin{array}{c} P_{0}\\ \vdots \\ P_{n} \end{array}\right],\;F=\left[\begin{array}{c} Q_{0}\\ \vdots \\ Q_{r} \end{array}\right],\;N=\left[\begin{array}{ccc} R_{0,p}\left({\bar{u}}_{0}\right) & \cdots & R_{n,p}\left({\bar{u}}_{0}\right)\\ \vdots & \vdots & \vdots \\ R_{0,p}\left({\bar{u}}_{r}\right) & \cdots & R_{n,p}\left({\bar{u}}_{r}\right) \end{array}\right]$$

Then minimize the expression in Equation (8) using least square method and the control points can be solved.

The basic framework of NURBS surface fitting from gridded data can be built on the foundation of NURBS curve fitting using the hierarchical fitting idea. That is, for a given set of points $Q=\{Q_{i,j},i=0,1,\ldots ,s;j=0,1,\ldots ,r\}$, points $Q_{i,j}$ are first fitted row by row with NURBS curves, and then the same operation is done on the resulting control points column by column to produce the final surface control points [18,21,28]. We call the fitting following row direction the *u*-direction fitting and that in column direction the *v*-direction fitting. In consideration of the application background of this paper, local modification (which often appears in the field of shape design) is not considered, and uniform weights are applied in the proposed method. The weights provide the possibility of shape modification after surface fitting, if needed [25].

### 2.3. Methods of NURBS Surface Fitting Based on Iterative Projection Optimization

Iterative projection optimization idea is not a novel choice among the methods of surface fitting. The major steps are: (1) project data points onto an appropriate base space, (2) do some operation, for example subdivision [41], based on the projection result, to get initial parameters, (3) get the knot vector and carry on surface fitting with the achieved parameterization result, (4) if the fitting quality doesn’t satisfy the given requirement, do iteration to modify the projection result and the parameterization. So when it is used for NURBS surface fitting, an iteration scheme and a projection algorithm are absolutely necessary.

In references [28,41,42], relevant research about point projection has been presented, and the basic process is usually performed in an iterative fashion, which generally has two steps: first to obtain a good start value; and second to operate the iteration until the distance converges to its minimum. With good start value, method based on Newton iteration is an excellent choice not only for its convergence speed but also for its stability. In this paper, approach based on Newton iteration is applied to calculate the projection points in NURBS curve and surface, and the basic process is provided in the next subsection.

#### 2.3.1. Calculating the Projection Point in Curve

A clear explanation of projection point used in this paper is listed here: C(u) is a given space curve, Q is a given point in the space, $C(u^{pro})$ is a point on the curve, if $\left|C(u^{pro})-Q\right|$ is the least distance between Q and C(u), then $C(u^{pro})$ is the projection point of Q on C(u) in this paper. 

**Figure 2:**
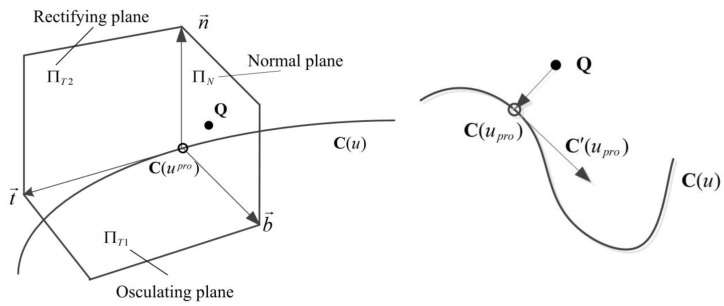
The projection point in a curve.

In the left picture of Figure 2, vectors $\vec{n}$, $\vec{t}$, $\vec{b}$ are respectively the principal normal vector, the tangent vector, and the binormal vector of curve C(u) at point $C(u^{pro})$, and plans $\Pi_{T1}$, $\Pi_{N1}$, $\Pi_{T2}$ are the corresponding osculating plane, normal plane and rectifying plane. By our definition of $C(u^{pro})$ as a point on curve closest to the given point Q, Q has to be in the curve’s normal plane at $C(u^{pro})$. Thereout, an objective function is given as follows to calculate the projection point $C(u^{pro})$ on curve C(u):

(9) $$f(u)=C^{\prime }(u)•(C(u)-Q)$$

where $C^{\prime }(u)$ is the derivative of curve C(u) with respect to parameter *u*.

According to the definition of normal plane, the projection point is the solution of f(u)=0. That is $C^{\prime }(u^{pro})•(C(u^{pro})-Q)=0$. Assuming that the start value $u_{0-pro}$ is already known, $u_{i-pro}$ is the result of *i*th Newton iteration, then the result of the (*i* + 1)th iteration is described as:

(10) $$u_{(i+1)-pro}=u_{i-pro}-f(u_{i-pro})/f^{\prime }(u_{i-pro})$$

The convergence criteria are usually Euclidean distance $\left|C(u_{i-pro})-Q\right|$ and cosine value of the angle between $C^{\prime }(u_{i-pro})$ and $C(u_{i-pro})-Q$. If $u_{i-pro}$ satisfies the given convergence condition, $u_{i-pro}$ is regarded as the $u^{pro}$, otherwise continue Newton iteration.

#### 2.3.2. Calculating the Projection Point in Surface

Suppose S(u,v) is a given surface, Q is a given point in the space, $S(u^{pro},v^{pro})$ is a point on the curve, if $\left|S(u^{pro},v^{pro})-Q\right|$ is the least distance between Q and S(u,v), then $S(u^{pro},v^{pro})$ is the projection point of Q on S(u,v) in this paper.

For surface S(u,v), only when Q is in the surface’s normal line at point $S(u^{pro},v^{pro})$, $\left|S(u^{pro},v^{pro})-Q\right|$ is the least distance between Q and S(u,v). Based on this, an matrix function f(u,v) which consists of two dot product functions $f_{1}(u,v)$ and $f_{2}(u,v)$, is built as follows:

(11) $$f\left(u,v\right)=\left[\begin{array}{c} f_{1}\left(u,v\right)\\ f_{2}\left(u,v\right) \end{array}\right]=\left[\begin{array}{c} S_{u}\left(u,v\right)\cdot \left(Q_{l,k}-S\left(u,v\right)\right)\\ S_{v}\left(u,v\right)\cdot \left(Q_{l,k}-S\left(u,v\right)\right) \end{array}\right]$$

where $S_{u}(u,v)$ and $S_{v}(u,v)$ are the first partial derivatives of S(u,v) in *u*-direction and *v*-direction, respectively. Then its projection point on S(u,v) is the point with a parameter pair $\left(u_{l,k},v_{l,k}\right)$ where $f(u_{l,k},v_{l,k})=0$ is met. Let matrix $\gamma_{i-pro}={\left[\Delta u_{i-pro},\Delta v_{i-pro}\right]}^{T}={\left[u_{(i+1)-pro}-u_{i-pro},v_{(i+1)-pro}-v_{i-pro}\right]}^{T}$ where $\left(u_{i-pro},v_{i-pro}\right)$ is the output of the *i*th Newton iteration and $\left(u_{(i+1)-pro},v_{(i+1)-pro}\right)$ is the output of the (*i* + 1)th iteration. $\gamma_{i-pro}$ can be gained in the *i*th iteration by solving below matric equation:

(12) $$\gamma_{i-pro}=-J_{i-pro}^{-1}f_{i-pro}$$

All the notations in Equation (10) are in matrix form, among which $f_{i-pro}$ is the value of object matrix function f(u,v) at $\left(u_{i-pro},v_{i-pro}\right)$, and $J_{i-pro}$ is the Jacobian matrix of f(u,v) evaluated at $\left(u_{i-pro},v_{i-pro}\right)$. Then the output of the (*i* + 1)th iteration is:

(13) $$\begin{array}{c} u_{(i+1)-pro}=u_{i-pro}+\Delta u_{i-pro}\\ v_{(i+1)-pro}=v_{i-pro}+\Delta v_{i-pro} \end{array}$$

Give convergence criterions based on Euclidean distance $\left|S(u_{i-pro},v_{i-pro})-Q\right|$, cosine value of the angle between $S(u_{i-pro},v_{i-pro})-Q$ and $S_{u}(u_{i-pro},v_{i-pro})$, and cosine value of angel angle between $S(u_{i-pro},v_{i-pro})-Q$ and $S_{u}(u_{i-pro},v_{i-pro})$. If $\gamma_{i-pro}$ satisfies the judgement criteria, $S(u_{i-pro},v_{i-pro})$ is the final projection point.

## 3. New Method of Surface Fitting from Scattered Data Points

As described in the introduction, for quasi-scattered data, a set of 3D points $Q=\{Q_{i,j},i=0,1,\ldots ,s;j=0,1,\ldots ,r_{i}\}$ can be regarded as the measured data of a surface S, where $r_{i}$ is the number of data points in the *i*th row, and for any i,j∈0,1,…,s, $r_{i}$ does not have to be equal to $r_{j}$. Assuming that points in the same row are captured from approximate iso-parametric or section curves, and each of these approximate lines never intersects with the others. Under this assumption, if curve $C_{l}(u)$ is the curve fitting from points $Q_{l,j},j=0,1,\ldots ,r_{l}$, and with fitting error ignored, it must be a part of **S** and will not intersect with other fitting curves, so in order to overcome the problem resulting from the randomness of point numbers in each row, a new method is given here: (1) first fits the points in Q row by row with NURBS curves, (2) resamples on the resulting curves $C_{l}(u)$, l=0,1,…,s and ensure equivalent number of points in each row, and (3) constructs a NURBS surface based on the resampled data $\bar{Q}$. Considering that no local modification is used in the proposed method, uniform weights are applied in both curve fitting and surface fitting, and for convenience of calculation, all weights are set equal to constant 1.

In the first and third part of this method, an iterative projection optimization idea is used, and it is not novel in NURBS object fitting. For example, in reference [24], a method based on iterative projection optimization is proposed, and it is outstanding in existing methods for its high accuracy and convergence speed when it comes to grid data. In [17], an error term called squared distance minimization (SDM) is introduced for planar curve fitting. It is defined by a curvature-based quadratic approximant of squared distances from data points to a fitting curve. A method based on this error term converges with fewer iterations comparing with methods based on point distance, but for space curves, the description of error term SDM may become too complicated. However, the curve fitted from measured points in one row of quasi-scattered data is generally a space curve, especially when the measured data is acquired by portable 3D coordinate measurement machines, so to be more general, point distance, which is widely used in practice for parametric curve and surface fitting, is selected in this paper.

**Figure 3:**
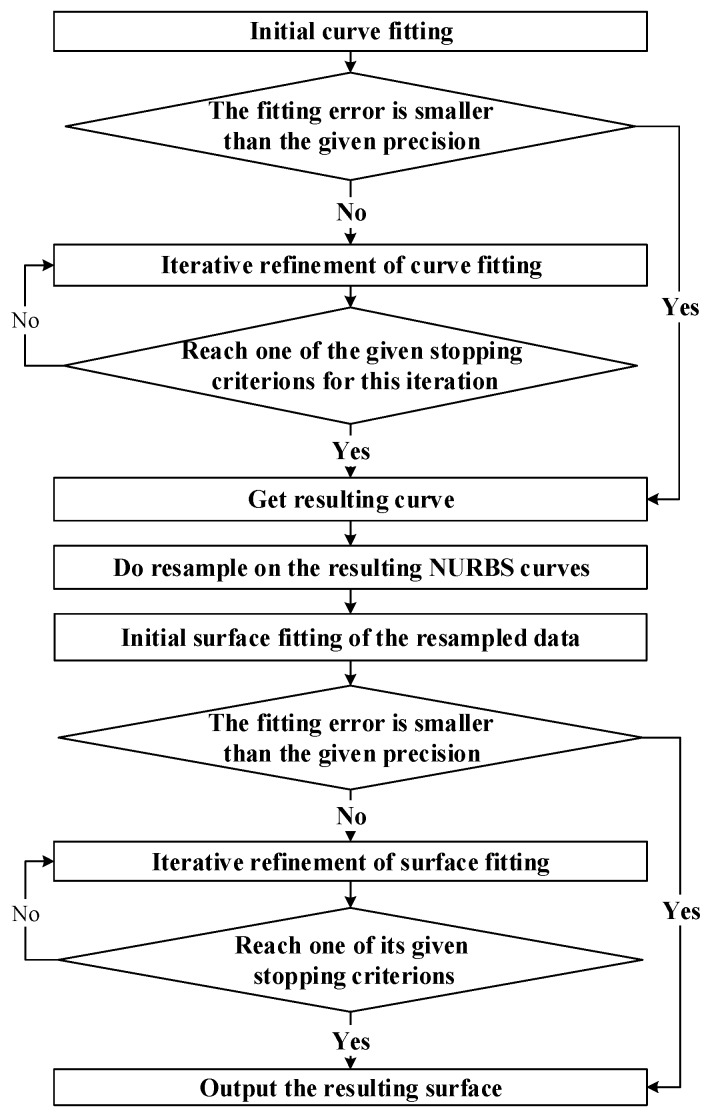
Framework of the method proposed in this paper.

In our method, new algorithms based on iterative projection optimization are given for NURBS curve and surface fitting, in which problems of data parameterization and point projection are merged together to reduce the time cost. A new resample approach which makes use of parameterization information of curve fitting and changes of curvature is proposed, which makes our method has advantages in accuracy and efficiency especially for surface fitting from quasi scattered data. The flowchart of this method is shown in Figure 3, and concrete algorithms and implementing procedures of three major parts: NURBS curve fitting, resampling and NURBS surface fitting are introduced separately in detail in Section 3.1, Section 3.2 and Section 3.3. 

### 3.1. NURBS Curve Fitting in Our Method

As can be seen in Figure 3, in order to construct a NURBS surface from quasi-scattered data $Q=\{Q_{i,j},i=0,1,\ldots ,s;j=0,1,\ldots ,r_{i}\}$, a set of NURBS curves must be obtained which accurately reflect the characteristics of measured object. In this part iterative projection optimization idea is used for its obvious advantages in simplicity and generality. The curve fitting for data points in each row is an iterative process where an initial fitting curve is firstly achieved in accordance with the thought provided in Section 2.2 and then followed by an iterative refinement based on projection. For the calculation of projection point, Newton iteration principle is used because with a good start value, it converges rapidly and stability. During one iteration of this algorithm, every $Q_{i,j}$ is projected onto the output of last iteration with its parameterization result in last iteration as the initial value of projection, and subsequently, reparameterize the data points based on the distribution of their corresponding projection points. With the given definitions of relevant symbols,

$Q_{l}$$Q_{l}=\{Q_{l,j},j=0,1,\ldots ,r_{l}\}$, data points in *l*th row of the given data Q$k_{\max}$The maximum number of iteration$\varepsilon_{0}$The given fitting precisionpCurve degreen+1The number of control points${\bar{U}}_{l}^{\left(k\right)}$${\bar{U}}_{l}^{\left(k\right)}=\{{\bar{u}}_{l,j}^{\left(k\right)},j=0,1,\ldots ,r_{l}\}$, parameterization result in *k*th iteration$U_{l}^{\left(k\right)}$The knot vector in kth iteration$P_{l}^{\left(k\right)}$$\left\{P_{l}^{\left(k\right)}=P_{l,j}^{\left(k\right)},j=0,1,\ldots r_{l}\right\}$, control points in *k*th iteration$C_{l}^{\left(k\right)}(u)$The resulting curve of *k*th iteration$u_{l,j}^{(k),\mathrm{pro}}$The resulting parameter of point projection from point $Q_{i,j}$ to curve $C_{l}^{\left(k\right)}(u)$${\bar{e}}_{l}^{\left(k\right)}$The average distance from any point in $Q_{l}$ to its projection point on $C_{l}^{\left(k\right)}(u)$$C_{l}(u)$The final fitting curve corresponding to $Q_{l}$

The detail process of curve fitting for $Q_{l}$ is as follows Algorithm 1:
**Algorithm 1**
k=0 (*initial curve fitting*)Do parameterization for $Q_{l}$ by Equation (5), save the result as ${\bar{U}}_{l}^{\left(0\right)}=\{{\bar{u}}_{l,j}^{\left(0\right)},j=0,1,\ldots ,r_{l}\}$Determine the knot vector $U_{l}^{\left(0\right)}$ based on ${\bar{U}}_{l}^{\left(0\right)}$ by Equation (6).Calculate control points $P_{l}^{\left(0\right)}$ according to Equations (7) and (8) with parameterization result ${\bar{U}}_{l}^{\left(0\right)}$ and knot vector $U_{l}^{\left(0\right)}$.Obtain the curve $C_{l}^{\left(0\right)}(u)$ based on $U_{l}^{\left(0\right)}$ and $P_{l}^{\left(0\right)}$. **for** $j=0,1,\ldots ,r_{l}$ **do**Do point projection from point $Q_{l,j}$ to curve $C_{l}^{\left(0\right)}(u)$: let ${\bar{u}}_{l,j}^{\left(0\right)}$ be the start value of Newton iteration, calculate the projection parameter $u_{l,j}^{(0),\mathrm{pro}}$ following the way presented in Section 2.3.1.**end for**Calculate the average distance ${\bar{e}}_{l}^{\left(0\right)}$**if** ${\bar{e}}_{l}^{\left(0\right)}\leq \varepsilon_{0}$ **do**$C_{l}(u)\leftarrow C_{l}^{\left(0\right)}(u)$**end the algorithm**k←k+1Update parameterization result ${\bar{U}}_{l}^{\left(k\right)}$ by projection result: ${\bar{u}}_{l,j}^{\left(k\right)}\leftarrow u_{l,j}^{(k-1),\mathrm{pro}}$Determine $U_{l}^{\left(k\right)}$ from updated ${\bar{U}}_{l}^{\left(k\right)}$ by Equation (6)Calculate control points $P_{l}^{\left(k\right)}$ according to Eq. (7)-(8) with ${\bar{U}}_{l}^{\left(k\right)}$ and $U_{l}^{\left(k\right)}$, and gain the curve $C_{l}^{\left(k\right)}(u)$**for** $j=0,1,\ldots ,r_{l}$ **do**Do point projection from point $Q_{l,j}$ to curve $C_{l}^{\left(k\right)}(u)$ by the presented way in Section 2.3.1 to get $u_{l,j}^{(k),pro}$, and in this projection step ${\bar{u}}_{l,j}^{\left(k\right)}$ is the start value of Newton iteration.**end for**Calculate the average distance ${\bar{e}}_{l}^{\left(k\right)}$.**if** ${\bar{e}}_{l}^{\left(k\right)}\leq \varepsilon_{0}$ or $k=k_{\max}$ **do**$C_{l}(u)\leftarrow C_{l}^{\left(k\right)}(u)$**end the algorithm**go back to step 13


**Figure 4:**
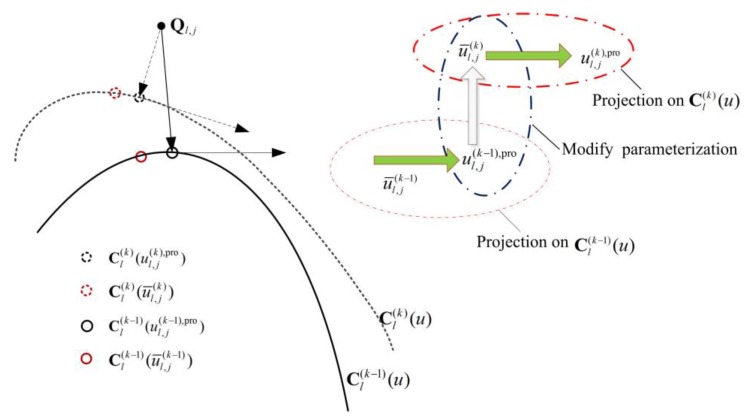
The process of iterative projection optimization during curve fitting.

In the *k*th iteration of the iterative projection optimization procedure, projection parameters gained from the (*k* − 1)th iteration are used to modify the previous parameterization in order to optimize the fitted curve. The projection point is calculated using Newton iteration approach presented in Section 2.3.1, with previous parameterization result used as the initial value. For example, in Figure 4, $C_{l}^{\left(k-1\right)}(u)$ is the fitted curve of the (*k* − 1)th iteration, ${\bar{u}}_{l,j}^{\left(k-1\right)}$ is the corresponding parameterization result of point $Q_{l,j}$, when do point projection from $Q_{l,j}$ to $C_{l}^{\left(k-1\right)}(u)$, parameter ${\bar{u}}_{l,j}^{\left(k-1\right)}$ is used as the initial value, and the resulting projection parameter is ${\bar{u}}_{l,j}^{(k-1),\mathrm{pro}}$. In the *k*th iteration, projection parameter ${\bar{u}}_{l,j}^{(k-1),\mathrm{pro}}$ is used to update the parameterization, after which corresponding parameter of point $Q_{l,j}$ is modified to ${\bar{u}}_{l,j}^{\left(k\right)}$, and the fitted curve is updated to $C_{l}^{\left(k\right)}(u)$. When projecting $Q_{l,j}$ to $C_{l}^{\left(k\right)}(u)$, ${\bar{u}}_{l,j}^{\left(k\right)}$ becomes the initial value.

The time complexity of the algorithm is $O(r_{l})$. Calculation of projection takes up the largest part of time cost. The authors sampled a row of 4000 data points from the model in example 1, and fit them using this algorithm. For every iteration when *k* > 0, more than 90% time is spent on the computation of projection. In this algorithm, parameterization result provides a good initial value for the calculation of projection point which makes Newton iteration method reach optimal projection result quickly. For the whole process, the maximum iteration number $k_{\max}=3$ is enough for most issues.

### 3.2. The Resample Approach in Our Method

In this paper, resampling is applied and it plays an important role in our method, because based on it the input quasi-scattered data can be converted into grid data. It is significant for the fitting accuracy and time cost of the whole method.

Today some well-known sampling approaches are usually uniform sampling, patch size based sampling, curvature-based sampling, the equal arc length sampling and the equal parameter sampling [43,44,45]. Uniform sampling first generates a straight line with sample nodes uniformly distributed on it, and then projects these nodes to the curve to get final sample points. It is not recommended when the curve is complex. Patch size-based sampling [43,44] divides the object into a set of ordinal units based on the knot vector. These units are ranked based on their own geometry sizes and points are distributed according to this ranking. That is, unit of the higher rank contains the more sample points, in which case, some important information in unit of very lower rank might be ignored. In curvature-based sampling, the sampling object is divided into independent units and for each unit the most critical points are first selected depending on its maximum and minimum curvature. Then more sample points are added to each unit based on the whole distribution of overall critical points, and the curvature variation can be reflected accurately by the resulting points. The equal arc length sampling, which adjusts the sampling density automatically from the curve slope, is also sensitive to curvature change. The equal parameter sampling, distributing sample points equally in parameter domain, is usually a nice choice when sample on parameter model with nonsignificant curvature changes.

As Figure 3 shows, in order to reconstruct a surface from a given set of quasi-scattered data $Q=\{Q_{i,j},i=0,1,\ldots ,s;j=0,1,\ldots ,r_{i}\}$, NURBS curves are obtained, and resampling is carried out on each of the resulting curves. During the process of resampling, three problems should be taken into consideration: (1) the number of sampling points is unknown, (2) guarantee that the number of resampled points in each row is the same, and (3) the resampled data should reflect the shape of surface without distortion. As mentioned above, if we just consider the first and the second problem, equal parameter sampling technique is enough and simple to realize. However, when the surface is complex in curvature, the resampling becomes troublesome through existing sample approaches.

Here a new resampling algorithm is given based on the parameterization result of curve fitting and changes of curvature. The basic idea is to sample more points in the area that curvature of curve changes rapidly and to sample less points in the area that the curve is gentle. The number of resampling points is set to be a little larger than the maximum number of original data points in each row. By this way, the resampling points not only preserve the information of original data completely but also include some important shape information acquired from the fitting curve. If the number is too large, additional resampling error may be introduced. 

In our method, resampling is conducted on the curve $C_{l}(u)$ fitted from each row of the measured data. When resampling on the parameter curve, the curvature variation with respect to the parameter should be gained first. In this paper, we add three more parameters between every two parameters in parameterization result, and calculate the curvature at both parameterization result and the added ones to get the curvature variation. For the reason that NURBS curve is a kind of spline curve, which means it is piecewise and segmented by knot vector, the calculated curvature has slight fluctuation in each segment. Therefore, GLPF (Gaussian low-pass filter) is adopted to smooth the curvature plot, and the parameters that achieve local maximum are saved as local peaks. 

Calculate the curvature integral value between any two adjacent parameters. For two adjacent parameters $u_{a}$ and $u_{b}$, $\left|u_{a}-u_{b}\right|\cdot \left|curvature(u_{a})+curvature(u_{a})\right|/2$ is used as the approximate curvature integral value. Because the parameterization in initial curve fitting is based on chord-length, the geometric meaning of the curvature integral is that it is approximately proportional to the angle that the tangent vectors have rotated from parameter $u_{a}$ to $u_{b}$. Adding the results together, we gain the whole integral value.

Next the whole integral value is divided into r parts (where *r* + 1 is the pre-set number of resampling point) equally, and the resulting unit controls the distribution of resampling points. That is, the curvature integral value between any two adjacent resampled parameters should be equal. By this way, it is guaranteed that there are more resampled points in the highly curved regions.

**Figure 5:**
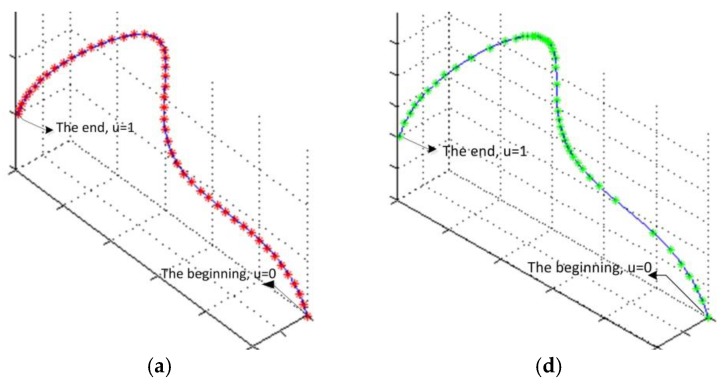

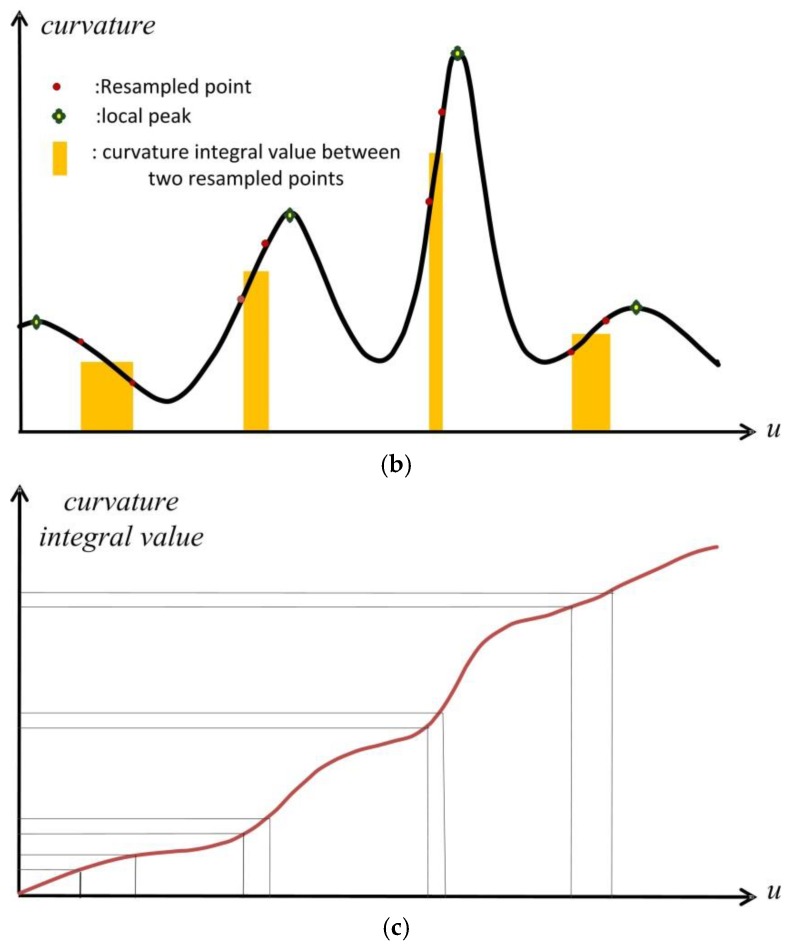
The process of resampling. (**a**–**d**) are respectively the measured data using equal parameter sampling technique, the curvature variation after GLPF, the curvature integral value, and the resampled points using the provided approach.

Figure 5 gives a clear explanation of the above process. Figure 5a–d are respectively the measured data using equal parameter sampling technique, the curvature variation after GLPF, the curvature integral value, and the resample result using the provided algorithm.

When resampling on the curves $C_{l}(u)$ l=0,1,…,s, definitions of the relevant symbols in resample are given as follows:
${\bar{U}}_{l}$The parameterization result of points in the lth row after curve fittingr+1The pre-set number of points resampled from each fitted curve$\bar{Q}$Save all the resampling points from curves $C_{l}(u)$, l=0,1,…,s${\overset{⌢}{u}}_{l,j}$Parameter corresponding to (j+1)th sampling point in the lth curve${\overset{⌢}{U}}_{l}$Save parameters of all sampling points of lth curve

The specific process of resampling is given as below Algorithm 2:

**Algorithm 2**
**for** l=0,1,…,s **do**
**for** $j=0,1,\ldots ,r_{l}-1$add parameters $({\bar{u}}_{l,j+1}-{\bar{u}}_{l,j})/4$, $({\bar{u}}_{l,j+1}-{\bar{u}}_{l,j})/2$, $3({\bar{u}}_{l,j+1}-{\bar{u}}_{l,j})/4$ into $\left[{\bar{u}}_{l,j},\;{\bar{u}}_{l,j+1}\right]$**end for**Save both parameterization result and the added parameters in array ACalculate curvature at every parameter of array AUse Gaussian low-pass filter to smooth the result in step 6, and save parameters where curvature achieves local maximums (local peak) in array BCalculate the curvature integral value between any two adjacent elements in A, and gain the whole integral value.Divide the whole integral value into r parts equally, denote as Δinte**for** j=1,…,r−1Calculate the parameter where curvature integral is Δinte⋅j, and save it as the *j*th resampled parameter ${\overset{⌢}{u}}_{l,j}$**end for****for** $j=0,1,\ldots ,r_{l}-1$**if** $u_{l,i}^{peak}$ exists in $\left({\overset{⌢}{u}}_{l,j},{\overset{⌢}{u}}_{l,j+1}\right)$ **do**use $u_{l,i}^{peak}$ to replace one of ${\overset{⌢}{u}}_{l,j},{\overset{⌢}{u}}_{l,j+1}$, the one nearer to $u_{l,i}^{peak}$ is replaced**end if****end for**Calculate point on $C_{l}(u)$ at parameters in ${\overset{⌢}{U}}_{l}$, and save the result in $\bar{Q}$.**end for**

With only consideration of the preservation of geometric properties, curvature-based sampling and the equal arc length sampling are all good choices, but it is extremely difficult for them to guarantee the number equality of resampled points for each row. The proposed algorithm overcomes this problem and keeps the advantage of curvature-based sampling. 

In this algorithm, parameterization result and its subdivided result (the added parameters in step 3) are used together to get the change of curvature. It is reasonable, because distribution information of the measured data is reserved in the parameterization result.

Curvature integral value in this algorithm is approximate to the included angle of tangent vectors. When resampling based on the unit integral value Δinte, for any two contiguous resampled points the included angle of tangent vectors almost keeps the same. The resulting points can reflect the curvature change of the curve and proposed algorithm keeps the advantage of curvature-based sampling. The time complexity of the algorithm is $O(r_{l})$.

### 3.3. NURBS Surface Fitting in Our Method 

In this section, the detailed algorithm of NURBS surface fitting from the resampled data $\bar{Q}=\{{\bar{Q}}_{i,j},i=0,1,\ldots ,s;j=0,1,\ldots ,r\}$ is given. In this algorithm hierarchical fitting idea, provided in Section 2.2, is applied. The whole framework is an iterative projection optimization process. As is known, when iteration projection idea is applied, in order to improve the fitting accuracy and arithmetic speed, advisable parameterization result and high-quality start value of point projection are especially important, in our method they are ensured by fusing point projection and data parameterization into each other during iteration with the same way as Section 3.1. Given the definitions of the relevant symbols:
$k_{\max}$The maximum number of iteration$\varepsilon_{0}$The given fitting precisionp,qSurface degree in *u*-direction and *v*-direction respectively${\bar{U}}^{\left(k\right)}$${\bar{U}}_{l}^{\left(k\right)}=\{{\bar{u}}_{l,j}^{\left(k\right)},j=0,1,\ldots ,r_{l}\}$, parameterization result in *k*th iteration$U^{\left(k\right)}$The *u*-direction knot vector in *k*th iteration${\bar{P}}^{\left(k\right)}$${\bar{P}}^{\left(k\right)}=\{{\bar{P}}_{i,j}^{\left(k\right)},i=0,1,\ldots ,s;j=0,1,\ldots ,n\}$ control points of *u*-direction in *k*th iteration ${\bar{V}}^{\left(k\right)}$${\bar{V}}^{\left(k\right)}=\{{\bar{v}}_{i,j}^{\left(k\right)},i=0,1,\ldots ,s;j=0,1,\ldots ,r\}$
*v*-direction parameterization result in *k*th iteration$V^{\left(k\right)}$The *v*-direction knot vector in *k*th iteration$P^{\left(k\right)}$$P^{\left(k\right)}=\{P_{i,j}^{\left(k\right)},i=0,1,\ldots n;j=0,1,\ldots ,m\}$, *v*-direction control points in *k*th iteration$S^{\left(k\right)}(u,v)$The resulting NURBS surface of *k*th iteration$\left(u_{i,j}^{(k),pro},v_{i,j}^{(k),pro}\right)$The resulting parameter of point projection from ${\bar{Q}}_{i,j}$ to $S^{\left(k\right)}(u,v)$${\bar{e}}^{\left(k\right)}$The average distance from any point in $\bar{Q}$ to its projection point on $S^{\left(k\right)}(u,v)$S(u,v)The final fitting surface corresponding to $\bar{Q}$


The details of this algorithm are as shown in the following Algorithm 3:

**Algorithm 3**
**begin iteration***k* = 0**while** (not termination condition) **do****for** l=0,1,…,s **do**Do parameterization for points in *l*th line by Equation (5), save the result in ${\bar{U}}^{\left(k\right)}$ denoted as ${\bar{u}}_{l,j}^{\left(k\right)},j=0,1,\ldots ,r$.**end for**Do average operation on ${\bar{U}}^{\left(k\right)}$: ${\bar{u}}_{j}\leftarrow \sum_{l=0}^{s}{\bar{u}}_{l,j}/\left(s+1\right),j=0,1,\ldots ,r$.Determine the knot vector $U^{\left(k\right)}$ following Equation (6) with ${\bar{u}}_{j},j=0,1,\ldots ,r$.**for** l=0,1,…,s **do**Do curve approximation for points in *l*th line following Equations (7) and (8), and save resulting control points in ${\bar{P}}^{\left(k\right)}$, denoted as ${\bar{P}}_{l,j}^{\left(k\right)},j=0,1,\ldots ,n$.**end for****for** j=0,1,…,n **do**Do parameterization for ${\bar{P}}_{i,j}^{\left(k\right)},i=0,1,\ldots ,s$ by Equation (5) and save the result in ${\bar{V}}^{\left(k\right)}$.**end for**Determine the knot vector $V^{\left(k\right)}$ based on ${\bar{V}}^{\left(k\right)}$ the same as that in *u*-direction.**For** j=0,1,…,n **do**Do curve approximation for points ${\bar{P}}_{i,j}^{\left(k\right)},i=0,1,\ldots ,s$ following the way in *u*-direction fitting, and get control points $P_{i,j}^{\left(k\right)},i=0,1,\ldots ,m$.**end for**Gain surface $S^{\left(k\right)}\left(u,v\right)$ based on $U^{\left(k\right)}$, $V^{\left(k\right)}$ and $P^{\left(k\right)}$.**for** i=0,1,…,s**for** j=0,1,…,r **do**Do point projection from point $Q_{i,j}$ to surface $S^{\left(k\right)}\left(u,v\right)$ to get $\left(u_{i,j}^{(k),pro},v_{i,j}^{(k),pro}\right)$ by the way presented in Section 2.3.2 with $\left({\bar{u}}_{i,j}^{\left(k\right)},{\bar{v}}_{i,j}^{\left(k\right)}\right)$ being the start value.**end for****end for**Calculate the average distance ${\bar{e}}^{\left(k\right)}$.**if** ${\bar{e}}^{\left(k\right)}>\varepsilon_{0}$ and $k<k_{\max}$ **do**k←k+1, update ${\bar{U}}^{\left(k\right)}$ with projection result, go back to step 7**else if** ${\bar{e}}^{\left(k\right)}\leq \varepsilon_{0}$ or $k=k_{\max}$ **do**$S(u,v)\leftarrow S^{\left(k\right)}(u,v)$****end if****
**end while****end iteration**

In this algorithm, the hierarchical fitting idea and iterative projection optimization are combined with each other, and during the iteration process, every point is projected onto the output of last iteration with its parameterization result in last iteration as the initial value of projection, and subsequently, re-parameterize the data points based on the distribution of their corresponding projection points. In our method uniform weights are used in the fitting process. Let *N* be the whole number of data points, the time complexity of this algorithm is O(N). In iterative projection methods the calculation of point projection is the most time-consuming part, while in this algorithm, the parameterization result of last iteration provides a suitable stat value with which the projection point is reached quickly by Newton method.

## 4. Experiment

In order to prove the validity of the proposed method, its performance has been tested on both simulation data and real-measured data. Some test results and a comparison with other previous methods are shown in this section, and a discussion about the factors that influence the fitting result is also presented in this chapter.

### 4.1. Illustrative Experiment

In this section, we do experiment on six different examples: three test problems from [24] (examples 3–5) and other three surfaces (examples 1, 2, 6). For each of example, a collection of grid data points is first fitted, and then the proposed method in Figure 3 is tested on a set of quasi scattered points. Results including fitting error, computation time, and information about final fitted surface are provided.

**Example** **1.** *A quadric surface. This surface is given by the following equation:*

$$z=\frac{y}{1+x^{2}/250000+y^{2}/250000},\;x,\;y\in \left[-500\;\mathrm{mm},500\;\mathrm{mm}\right]$$

**Figure 6:**
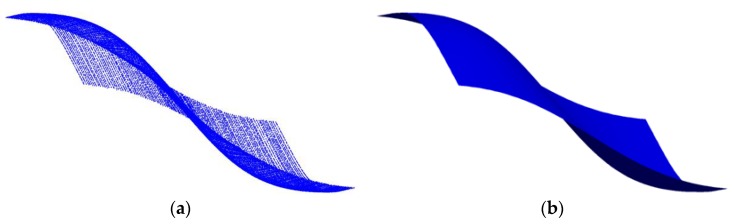
Surface fitting of a quadric surface: (**a**) the data points chosen randomly from grid data; (**b**) the fitting surface.

In this example, method for grid data fitting is tested on a collection of 334×334 data points, and a (4, 4) order fitting surface with 65×65 control points is obtained in 2.3 min, for which the average fitting error is $1.61\times 10^{-3}\;\mathrm{mm}$ and the maximum error is $2.97\times 10^{-3}\;\mathrm{mm}$. We chose 97,612 points randomly from the grid data, test the proposed method in Figure 2 on the resulting data, and a (4, 4) order NURBS surface of 65×65 control points is achieved in 3.1 min with an average fitting error of $1.67\times 10^{-3}\;\mathrm{mm}$, a maximum fitting error of $3.01\times 10^{-3}\;\mathrm{mm}$. Figure 6 presents the randomly chosen data and its fitting surface.

**Example** **2.** *A hat surface. This surface is given as follows:*

$$z=\frac{100\sin\sqrt{x^{2}/2500+y^{2}/2500}}{\sqrt{x^{2}/2500+y^{2}/2500}},\;x,\;y\in \left[-400\;\mathrm{mm},400\;\mathrm{mm}\right]$$

**Figure 7:**
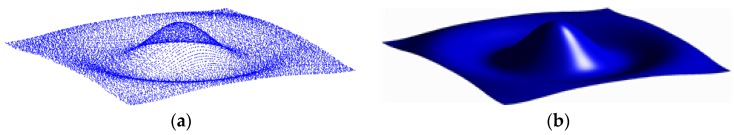
Surface fitting of a hat surface: (**a**) the scattered data chosen randomly from grid data; (**b**) the fitting surface.

In this example, a set of 267×267 data points is fitted with a (4, 4) order NURBS surface with 68×68 control points first. The average and maximum fitting error are $1.65\times 10^{-3}\;\mathrm{mm}$ and $3.34\times 10^{-3}\;\mathrm{mm}$ respectively, and the time cost is 1.7 min. For a collection of 53,367 scattered points chosen from the grid data, a (4, 4) order NURBS surface is obtained using the proposed method, with an average error of $1.69\times 10^{-3}\;\mathrm{mm}$ and a maximum error of $3.35\times 10^{-3}\;\mathrm{mm}$ in 2.4 min. The randomly chosen data and its corresponding fitting surface are displayed in Figure 7.

**Example** **3.** *A shell surface. This parametric surface is given as follows:*

$$\{\begin{array}{c} x=\frac{1}{5}\left(1-\frac{v}{2\pi }\right)\cos\left(2v\right)\left[1+\cos\left(u\right)\right]+\frac{1}{10}\mathrm{co}\left(2v\right),\\ y=\frac{1}{5}\left(1-\frac{v}{2\pi }\right)\sin\left(2v\right)\left[1+\cos\left(u\right)\right]+\frac{1}{10}\sin\left(2v\right),\\ z=\frac{v}{2\pi }+\frac{1}{5}\left(1-\frac{v}{2\pi }\right)sin\left(u\right), \end{array}\begin{array}{ccc} & u,v\in \left[0,2\pi \right] & \end{array}$$

**Figure 8:**
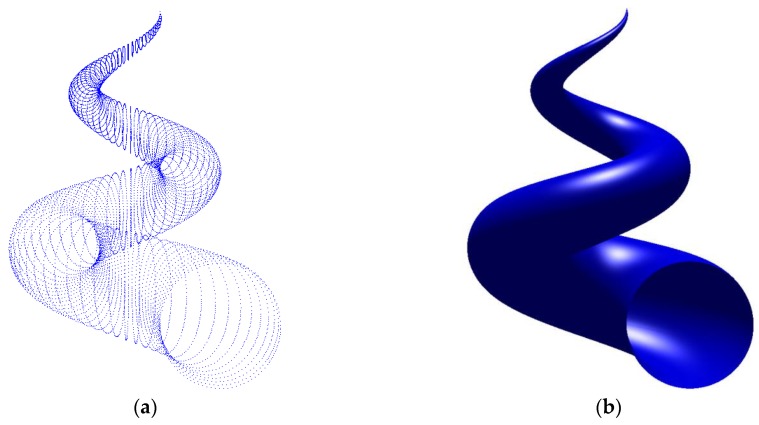
Surface fitting of a shell surface: (**a**) the data chosen randomly from grid data; (**b**) the fitting surface.

For this shell surface, grid data of 45×55 points is fitted, and a (4, 4) order NURBS surface is obtained in 87 s with an average error of $5.92\times 10^{-7}$. We conduct our method provided in Figure 3 on 2102 scattered points, chosen randomly from the grid one, and a (4, 4) order NURBS surface with 11×14 control points is accepted as the final result after 2.1 min, with the average error and the maximum error of $6.63\times 10^{-7}$ and $3.83\times 10^{-6}$, respectively. Figure 8 shows the distribution of the randomly chosen points and the corresponding fitting surface.

**Example** **4.** *A posit surface. This posit surface is given in parametric form as follows:*

$$\{\begin{array}{c} x=A\cos\left(B+u\right)\left(2+\cos\left(v\right)\right),\\ y=C\cos\left(D-u\right)\left(2+E\cos\left(F+v\right)\right),\\ z=E\cos\left(F+u\right)\left(2+G\cos\left(H-v\right)\right), \end{array}\begin{array}{cc} & u,v\in \left[0,2\pi \right] \end{array}$$

*where A = 0.655866, B = 1.03002, C = 0.74878, D = 1.40772, E = 0.868837, F = 2.43773, G = 0.495098, H = 0.377696.*

**Figure 9:**
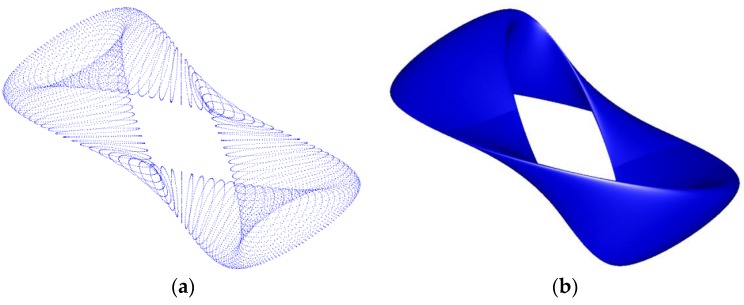
Surface fitting of a posit surface: (**a**) the data chosen randomly from grid data; (**b**) the fitting NURBS surface.

For this non-zero genus surface, method for grid data is applied on a set of 100×105 grid points, and the proposed method is carried out on 8725 points chosen randomly from the grid data. For the former one, the average error, the maximum error and the time cost are $1.1\times 10^{-6}$, $3.24\times 10^{-5}$ and 3.8 min successively. And for the scattered data, an average error of $2.35\times 10^{-6}$ is obtained in 5.2 min, where a (4, 4) order NURBS surface with 21×21 control points is accepted as the final resulting surface, the maximum error of which is $3.51\times 10^{-5}$. Figure 9 provides the randomly chosen data and the fitted surface.

**Figure 10:**
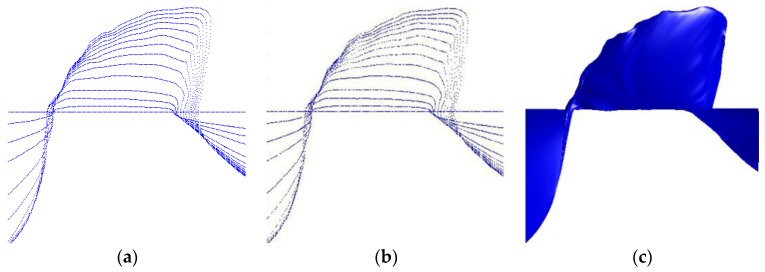
Surface fitting of a pump surface: (**a**) the original grid data; (**b**) the randomly chosen data; (**c**) the fitting NURBS surface.

**Example** **5.** *A pump surface. For the convenience of comparison, here the real measured data points provided in [24] is adopted. It is a grid data with 14 rows and the point number of every row is 370. Every data point is consisted of three variables: rotation speed, flow and moment, measured from the Mashan Pumped Storage Power Station in China [24], and the problem is to construct a model surface which reflects the concrete mathematical relations of the three variables. For this original data, method in [24] can reach a fitting accuracy of* $8.7\times 10^{-4}$ *in 1.3 min. Then for every row, we reject a part of points randomly and get a set of 4800 scattered data, from which a (5, 4) order NURBS fitted surface with* 97×4 *control points is achieved in 1.8 min by the proposed method, where the average error is* $8.9\times 10^{-4}$ *and the maximum error is* $1.71\times 10^{-3}$*. Figure 10 presents the original data, the randomly chosen data and the fitting surface.*

**Figure 11:**
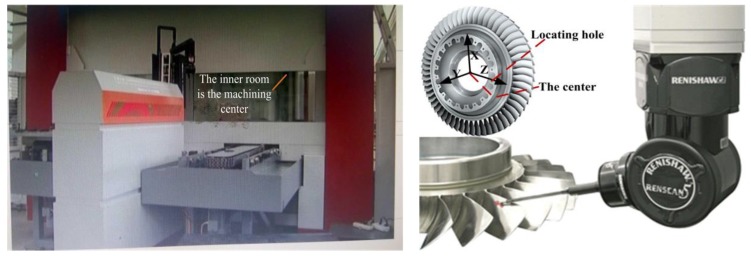
The applied coordinate measuring system: the left picture is the scene photo; and the right picture is the measurement on the workpiece.

**Example** **6.** A blade surface. In this example, data points are measured from a blade of aircraft engine by an in-situ automatic measuring machine system [46]. The left picture of Figure 11 is the scene photo (The inner room is the machining center). When the machining of one blade of the blisk is finished, the measuring machine rotates into the machining center to do measurement of the machined blade just like what is shown in right picture of Figure 11. The applied measuring probe is RSP2 probe, and it is installed on the Renscan5^TM^ probe body. The maximum permissible error value of REVO system is no more than 1 μm, and the uncertainty of the whole measuring system is less than (10 + L/30) μm (where L is the length value of the measured object, L: mm).

The measuring machine system consists of six relative motion parts, as a result, six coordinate systems from the tip center to the machine zero position are established based on the quasi-rigid-body model for building the machine coordinate system. The workpiece coordinate system is established based on the center circle of the blisk and the locating hole, as shown in the right picture of the Figure 11. The center of the circle is used as the original point of the coordinate system. The Z-axis of the coordinate system is determined by the line that connects the centers of the circle and the locating hole. The Y-axis is determined by the vertical axis of the center circle, and then X-axis is determined by the cross product of Y-axis and Z-axis.

**Figure 12:**
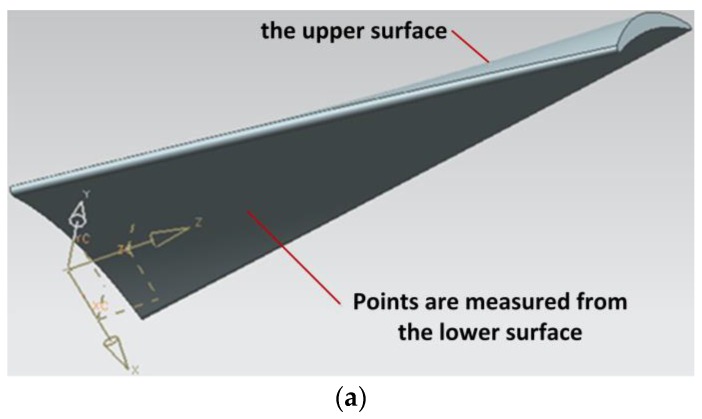

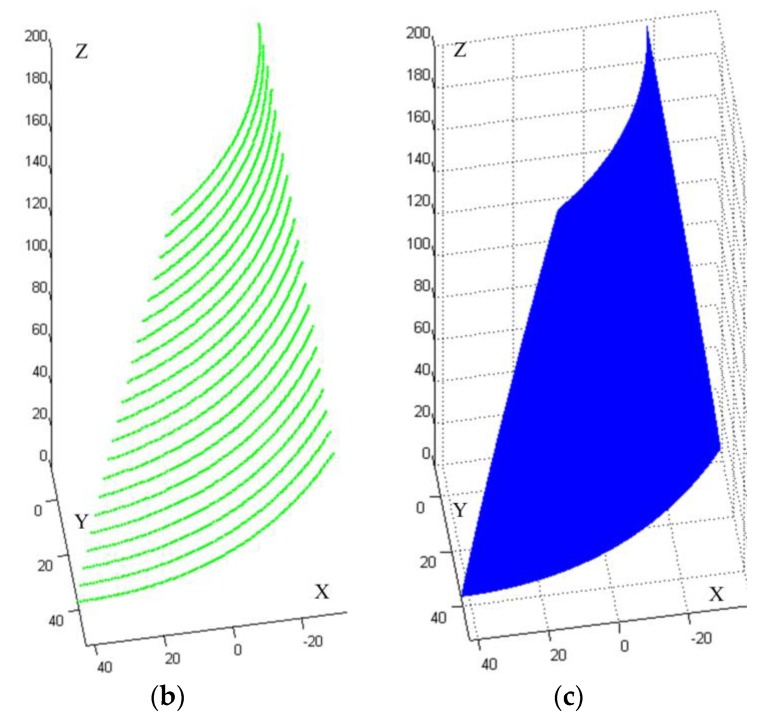
Surface fitting of a blade surface: (**a**) a diagram of the measurement; (**b**) the original grid data; (**c**) the randomly chosen data; (**c**) the fitting NURBS surface.

Twenty one sections of the blade are determined before the measuring process firstly. Because the twist angle of the blade is as much as 64° and the space between two adjacent blades is so small, one blade is divided into two surfaces (the upper surface and the lower surface). For every surface, do the measurement following these determined sections one by one. The data that used is quasi scattered data consisting of 21 rows. The maximum number of data points in a row is 118 and the minimum is 97. The whole data number is 2255. By the proposed method, a (4, 4) order NURBS surface of 24×9 control points is fitted in 17 s with an average fitting error of $1.22\times 10^{-3}\;\mathrm{mm}$, a maximum fitting error of $2.27\times 10^{-3}\;\mathrm{mm}$. Figure 12 presents the data and its fitting surface.

According to the above fitting results, our method performs well for scattered data chosen randomly from grid data, and the fitting results of these scattered data and grid data are in the same level with regard to both fitting error and time cost. For all experiments, the computations in this paper have been performed on a PC equipped with a core processor operating at 2.7 GHz with 2 GB of RAM. Calculation of projection takes up the dominant part of time cost. The source code has been implemented in VC++, while the resulting pictures are constructed in Matlab with the obtained control points, knot vectors, and the orders.

### 4.2. Comparison with Other Approaches

As stated, the proposed fitting method by curvature based resampling presents a good performance for surface fitting of problems analyzed above. Compared with other methods presented in the literature, it outstands when the fitting data is scattered, especially in terms of time cost. In order to support this claim, here we do a careful comparison with five other methods found in literature [8,18,23,24,33]. Table 1 shows the comparison results, including the average fitting error and the time cost. 

The method given by Ma in [8], projects points to a base surface created from approximate boundary points or curves to do parameterization. However, it works properly only for simple surface with no self-intersecting, and hard to get a high fitting accuracy. As shown in the comparison results, it has no advantage on time cost and fitting accuracy when used for quasi scattered data fitting. 

In [18], the rows are approximated independently of one another, and new knots are added into the knot vector during the curve fitting, then the merged knot vector is used for the fitting of the next row. This make the method face the problem of nodes redundancy. And in practice, it is often happens especially when the number of data points is large and the point distributions of different rows change a lot.

Methods in [23], given by Gálvez, based on particle swarm optimization, can achieve perfect accuracy for examples 1–5, but its complexity and computational time are too hard to handle. Compared with it, our method has a great advantage on time cost.

For the same quasi scattered points of examples 1–5, methods provided in [24,33] all report reasonable fitting accuracy at the same level with ours, but they all lose the competition on time expenditure.

To summarize, when used for quasi scattered data fitting, our method reaches enough accuracy with the lowest time cost compared with previous fitting approach.

### 4.3. Robustness

**Figure 13:**
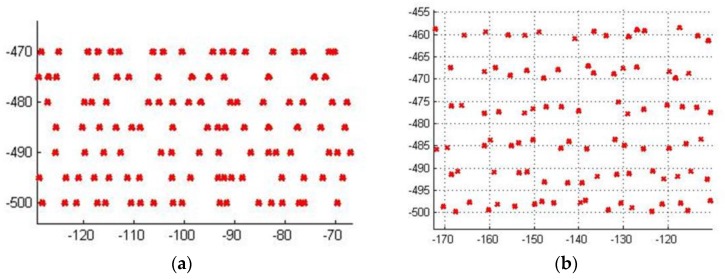
Points distribution on the (*u*, *v*) domain when sampling along *u*-section line: (**a**) the case when *u*-fluctuation is equal to zero; (**b**) the case when *u*-fluctuation ranges from −1.5 to 1.5 randomly. In this picture, v direction is the horizontal direction, and u direction is the vertical direction.

In order to test the robustness of the proposed method, we conduct the method on several data with varying degrees of scatterness acquired from examples 1 and 5. In example 1, model surface is presented in the implicit form of z=z(x,y), while if we let $x=v{,}_{}y=u$, it changes to an equivalent parametric form $x=x(u,v)=v{,}_{}y=y(u,v)=u{,}_{}z=z(u,v)$. To describe the experiment process on example 1 clearly, row-spacing, *u*-section line, *v*-interval, *u*-fluctuation and additional error introduced by resampling are introduced, and the special meanings of them are given as follows:

- *u-section line*: Points in such a section line which have the same coordinate in *u* direction.
- *row-spacing*: For two adjacent *u*-section lines, coordinate difference in *u* direction is row-spacing. If a set of section lines distribute uniformly, row-spacing is a constant value, otherwise it varies in a certain range.
- *v-interval* and *u-fluctuation*: When sampling along a *u*-section line, coordinate difference in *v* direction of two adjacent sample points is *v*-interval and the distance in u direction from a data point to the section line is *u*-fluctuation. If *u*-fluctuation is equal to zero anywhere, the sampling data points distribute as shown in Figure 13a, otherwise as shown in Figure 13b which is more similar to real measured data.
- *additional error introduced by resampling:* If the average distance between measured data and the final fitting surface S(u,v) is denoted as e called the average fitting error, and that between resampled data and S(u,v) is denoted as $\bar{e}$ then the absolute difference $\left|e-\bar{e}\right|$ is the additional error introduced by resampling.

**Figure 14:**
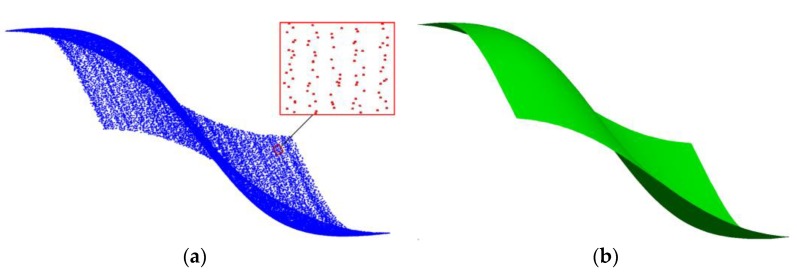
Distribution of data points corresponding to the 11th experiment in Table 2: (**a**) distribution in domain of quasi scattered data points; (**b**) the fitting surface.

After multiple tests on example 1, results of 12 representative experiments are listed in Table 2. Figure 14 gives the distribution of data points corresponding to the 11th experiment this table. In this table, *v*-interval, row-spacing, and *u*-fluctuation are used together to represent the scatterness of data distribution, the average error and additional error introduced by resampling are listed as the fitting results. 

In example 5, the real measured data provided in [24] is adopted as the original measured data. For this data, method in [24] can reach a fitting accuracy of $8.8\times 10^{-4}$. Here, several sets of scattered data points are acquired on the basis of the measured data. That is, each data point of the original measured data is reserved or rejected by random gating sampling, and the number of points in each row of the resulting data varies in a range randomly. Experiments are conducted on both the original measured data and the resulting scattered data to verify the robustness. Table 3 lists experimental results of the original measured data and four sets of resulting scattered data.

By comparing the different experimental results in Table 2, we know that the fitting precision of our method has inverse relation to the *u*-fluctuation and the row-spacing. Resample is brought in to implement data transformation in our method, in order to quantify its impaction on final fitting precision, additional error brought by resample (AER) is calculated in Table 2. And as shown in the results, it is always zero when sampling along sections strictly (the *u*-fluctuation is zero), in which case resample of course has no impact on fitting precision but this is not likely to happen in actual measurement. In general, it is not zero but small enough to be ignored compared with average fitting error (AFE). 

For the real-measured data points, the fitting accuracy of method provided in [24] based on iterative projection optimization is $8.8\times 10^{-4}$ and our method can reach a fitting accuracy of $8.7\times 10^{-4}$ in few seconds, what’s more, the fitting accuracy of our method nearly unchanged after removing some of the data points randomly as listed in Table 3, which illustrates the robustness against data missing and demonstrates that our method can be better applied in surface fitting of real-measured quasi-scattered points.

In order to test the performance in circumstance that there are areas with densely packed rows and areas with large distance between individual rows, we set rowing-spacing to be different value in different parts of the surface in example 2. When areas with densely packed rows (rowing-spacing is set to be 0.5~1) exist in the central part (180<x<220) where curvature change highly and areas with large distance (rowing-spacing is controlled between 10~12.5) exist in the part that the curve change gently (30<x<100), the average fitting error is $6.53\times 10^{-3}\;\mathrm{mm}$. While when the areas with large distance exist in the central part, the average fitting error is $8.61\times 10^{-2}\;\mathrm{mm}$. So it is the distribution of measured data not the distribution of rows that affect the final fitting result.

## 5. Conclusions

In this paper, a new method of NURBS surface fitting from quasi-scattered data is proposed. It consists of three parts: NURBS curve fitting for each row of the original data, curvature-based resampling on resulting curves and NURBS fitting for the resampled data. The proposed resampling approach is mainly on the basis of the parameterization result of the preceding curve fitting, and integrates curve curvature and local peaks. In proposed resampling approach, parameterization result and its subdivided result are used together to get the curvature variation. Chord length parameterization, which is close to arc length parameterization, is used in this paper because quasi scattered is easy to be organized into rows. Therefore, the parameterization result reserves the distribution information of original measured data, which can reflect the key geometric information of the curve. Besides, in this approach curvature is integrated along parameter u to get the curvature integral unit that used to control the distribution of the resampled data. It is easy to operate. And as the experimental results shown, it almost introduces no additional error in subsequent surface fitting. Iterative projection optimization idea is applied in the fitting process, but in our method parameterization results are used as the start value of point projection, so that problems of parameterization and calculation of projection are boned together, which makes the iteration reach reasonable results quickly and precisely. For quasi scattered data, the proposed method is efficient and superior to previous methods especially in the time coat. Experimental results described above have proved this. 

## Figures and Tables

**Table 1:** This is a comparison of proposed method with four other alternative methods. NDP presents the number of data points. AFE is the average fitting error, and TC denotes the time cost.

Method	Quadric Surface NDP: 97612 AFE, TC	Hat Surface NDP: 53367 AFE, TC	Shell Surface NDP: 2102 AFE, TC	Posit Surface NDP: 8725 AFE, TC	Pump Surface NDP: 4800 AFE, TC	Blade Surface NDP: 2255 AFE, TC
[8] 1995, Base surface parameterization with LSQ	$1.14\times 10^{-4}$, several h	$2.12\times 10^{-4}$, several h	failed	failed	$1.47\times 10^{-2}$, 94.7 min	$1.83\times 10^{-4}$, 30–50 min
[18] 2000, Surface fitting based on hierarchical fitting and merged knot vector	failed	failed	$4.51\times 10^{-4}$, 7.6 min	$1.35\times 10^{-3}$, 14 min	$4.91\times 10^{-3}$, 8.4 min	$2.58\times 10^{-4}$, 5.9 min
[23] 2010, Particle swarm optimization	$7.0\times 10^{-7}$, >100 min	$7.64\times 10^{-7}$, >100 min	$8.72\times 10^{-14}$, 35–50 min	$2.81\times 10^{-12}$, >100 min	$1.63\times 10^{-3}$, 11.4 min	$1.97\times 10^{-6}$, 7.9 min
[33] Iteration genetic algorithm with LSQ fitting	$1.79\times 10^{-5}$, >100 min	$8.7\times 10^{-5}$, >100 min	$2.83\times 10^{-6}$, >100 min	$9.1\times 10^{-6}$, >100 min	$7.43\times 10^{-3}$, >100 min	$4.11\times 10^{-6}$, 16.4 min
[24] 2010, projected optimization and iteration	$4.27\times 10^{-5}$, 18.3 min	$5.74\times 10^{-5}$ 15.9 min	$3.81\times 10^{-6}$, 11.2 min	$1.80\times 10^{-6}$, 45.7 min	$3.42\times 10^{-3}$, 32.3 min	$6.72\times 10^{-6}$, 2.1 min
Our method	$1.67\times 10^{-6}$, 3.1 min	$1.69\times 10^{-6}$, 2.4 min	$6.63\times 10^{-7}$, 2.1 min	$2.35\times 10^{-6}$, 2.8 min	$8.9\times 10^{-4}$, 1.8 min	$1.22\times 10^{-6}$, 17 s

**Table 2:** Experiment results of example 1. RND and NDP are the row number of data, the number of data points, respectively. RS, VIT and UFL are row-spacing, *v*-interval and *u*-fluctuation, successively. AFE and AER are respectively the average fitting error and the additional error introduced by resampling. In the RS, VIT and UFL columns, a~b means ranging from “a” to “b” randomly.

RS/mm	RND	VIT/mm	UFL/mm	NDP	AFE/mm	AER/mm
3	334	3~4.5	0.0	95,211	0.00169	0.00000
3	334	3~6	0.0	86,606	0.00171	0.00000
3	334	3~6	−0.6~0.6	86,606	0.00193	0.00001
3	334	3~6	−1.2~1.2	86,606	0.00226	0.00003
3~6	222	3~6	−0.6~0.6	57,520	0.00255	0.00003
3~6	222	3~6	−1.2~1.2	57,520	0.00316	0.00005
5	200	3~6	0.0	51,775	0.00254	0.00000
5	200	5~10	0.0	39,199	0.00304	0.00000
5	200	3~6	−1.0~1.0	51,775	0.00310	0.00006
5	200	3~6	−1.5~1.5	51,775	0.00379	0.00005
5~7.5	161	3~6	−1.0~1.0	41,668	0.00407	0.00006
5~7.5	161	3~6	−1.5~1.5	41,668	0.00484	0.00005

**Table 3:** Experiment results of example 5. Max-NRP and Min-NRP are the maximum and minimum number of data points in each row of the scattered data. The meanings of the other abbreviations are the same as Table 2.

RND	NDP	Max-NRP	Min-NRP	AFE
14	5180	370	370	$8.7\times 10^{-4}$ ^1^
14	4800	367	311	$8.9\times 10^{-4}$
14	4389	353	265	$8.9\times 10^{-4}$
14	4088	365	228	$9.0\times 10^{-4}$
14	4011	360	192	$8.9\times 10^{-4}$

^1^ Experiment of the first row is conducted on the original gridded data, and experiments of other rows are conducted on quasi scattered data.
